# Influence of methylphenidate on brain development – an update of recent animal experiments

**DOI:** 10.1186/1744-9081-2-2

**Published:** 2006-01-10

**Authors:** Thorsten Grund, Konrad Lehmann, Nathalie Bock, Aribert Rothenberger, Gertraud Teuchert-Noodt

**Affiliations:** 1 Department of Neuroanatomy, Faculty of Biology, University of Bielefeld, Universitätsstr. 25, D-33615 Bielefeld, Germany; 2 Child and Adolescent Psychiatry, University of Göttingen, von-Siebold-Strasse 5, D-37075 Göttingen, Germany

## Abstract

Methylphenidate (MPH) is the most commonly used drug to treat attention deficit/hyperactivity disorder (ADHD) in children effectively and safely. In spite of its widespread application throughout one of the most plastic and sensitive phases of brain development, very little is known to date about its long-term effects on brain structure and function. Hence, this short review updates the influence of MPH on brain development, since recent human and animal studies suggest that MPH alters the dopaminergic system with long-term effects beyond the termination of treatment.

Animal studies imply that the effects of MPH may depend on the neural responder system: Whereas structural and functional parameters are improved by MPH in animals with psychomotor impairments, they remain unaltered or get worse in healthy controls. While recent behavioural studies do not fully support such a differential effect of MPH in ADHD, the animal studies certainly prompt for further investigation of this issue. Furthermore, the abuse of MPH, when (rarely) intravenously applied, may even impair the maturation of dopaminergic fibres in subcortical brain areas. This argues for careful clinical assessment and diagnostics of ADHD symptomatology not only in conjunction with the prescription of MPH. Hence, one should be assured that MPH is only given to children with clear ADHD symptomatology leading to psychosocial impairment. The animal data suggest that under these conditions MPH is supportive for brain development and the related behaviour in children with ADHD.

## Background and rationale

Attention deficit/hyperactivity disorder (ADHD) is one of the most common behavioural disorders in childhood and may persist into adulthood. According to conservative estimates, its prevalence is around 3–5% [1]. Including subclinical cases with less stringent criteria used the percentage may rise up to 17% [2]. Although the aetiology of the disorder is not yet fully understood, a high heritability with vulnerability genes as basis seems to explain most of the behavioural variance, although obstetric complications and psychosocial adversity may play a role, too [see recent reviews by [1,3,4]]. In the last years, models of the neurobiological background of ADHD have become both more substantial and more complicated by a wealth of studies which showed that several, rather than one or a few, neuronal systems are likely to be involved, and that circuits contributing to motor regulation, executive functions, attention and delay of reinforcement, i.a., may be impaired in ADHD patients [rev. in [1,4,5]]. Animal models have played an important role in gathering this knowledge [rev. in [6,7]].

Methylphenidate (MPH) is the most widely used drug and the golden standard to treat ADHD [rev. [1,8]]. Its efficacy and safety has been documented in many studies [9] However, there is still a gap of knowledge concerning the influence of MPH on brain development and its long-term effect on brain structure and function.

Childhood and adolescence are a highly plastic and sensitive period of brain maturation, during which environmental and pharmacological influences exert strong effects on neural structure and function [see [10,11] for rev.]. Especially, cognitive, motivational and emotional functions mature intensively during this period of life. Such functions are subserved by brain areas that are characterised by a selective innervation of dopamine (DA), i.e. the prefrontal cortex (PFC), nucleus accumbens (NAc) and amygdala. The DAergic innervation of these areas matures late and passes through a phase of drastic anatomical and physiological upheaval during periadolescence [11-13]. Thus, MPH, considered to act as a DA agonist by blocking the DA and, to a weaker extent, noradrenaline transporters [14-16] might influence this process. Although no neurotoxic action of MPH has been reported so far [17-19], it is quite likely that pharmacological interference with the maturing DA system may lastingly change the developmental outcome [8,20,21].

Unfortunately, to date, there exist only few studies investigating the long-term plastic neuronal effects of MPH. But it is known that neurotransmitters and their agonists exert a strong morphogenetic influence on single neurons and nervous tissues [22-26], and even small environmental events can lastingly shape the brain if applied over a longer period [27-31](Lehmann, Grund et al., unpublished observations). Indeed, some studies have already shown that early treatment with clinical doses of MPH persistently changes DAergic parameters in rodents [20,32-34]. We therefore dedicate this mini-review to the behavioural and neurobiological long-term effects of MPH in humans and experimental animals.

### Dopamine function and dysfunction in ADHD

**Figure 1:**
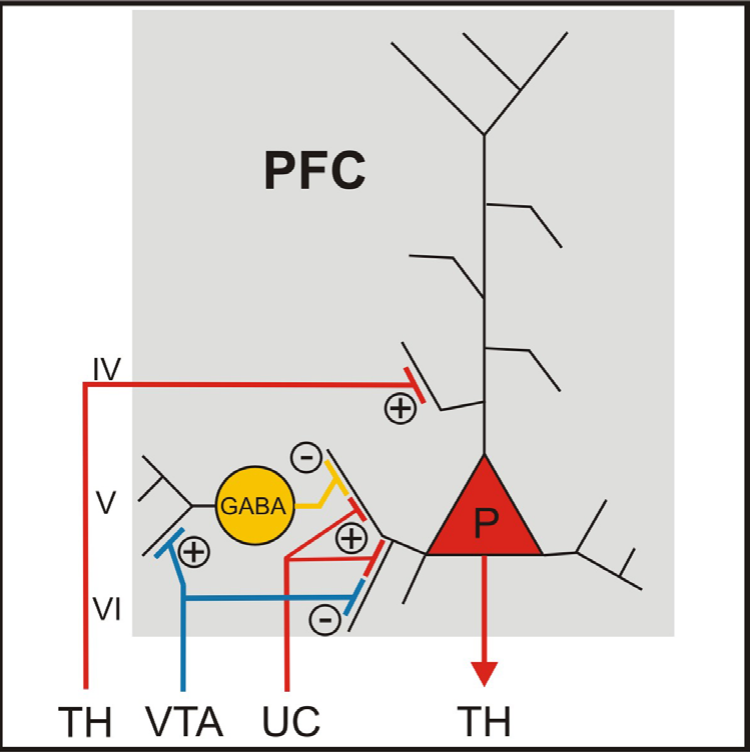
**Neuronal connections of Prefrontal Cortex**. Dopamine (DA) fibres arising in the ventral tegmental area terminate on GABAergic interneurons and glutamatergic pyramidal cells in the PFC. DA serves as a switch between cortical (with low DA transmission) and thalamic input (with high DA transmission) [117,118]. +-signs signify an excitation and – signs signify an inhibition. IV, V and VI = layer IV, V and VI; P = pyramidal cell; PFC = prefrontal cortex; TH = thalamus; UC = u-shaped cortical connections; VTA = ventral tegmental area.

As an indirect DA agonist, MPH presumably enhances DAergic transmission in the very same brain areas that play such an important role for cognition and emotion, and two of them – the PFC and the NAc – are considered to be principally involved in the aetiology of ADHD. Genetic research and in vivo imaging observations have put the focus on DA dysfunction in ADHD by documenting increased dopa decarboxylase activity in the midbrain [35], decreased sensitivity of the DA receptor type 4 and increased density of the DA transporter (DAT) in the striatum/NAc [36-42]. In the PFC, there is a reduced DA storage in ADHD patients [43], and it has been shown that MPH increases the extracellular DA concentration in the PFC [44,45]. This cannot, however, be achieved in a straightforward way, since neither DAT nor D2 receptors are present in detectable or even sufficient amounts in the PFC [46-49]. Instead, it has been shown that MPH blocks not only the DAT, but also the noradrenaline transporter (NAT) [15], and that DA is cleared by the NAT in the PFC [50]. Since DA serves as a switch between cortical input into the PFC (with low DA transmission) and thalamic input (with high DA transmission, fig. 1), the functional consequence will be a behaviour that is more driven by information coming from non-cortical regions, rather than by intrinsic cortical information [109,110].

**Figure 2:**
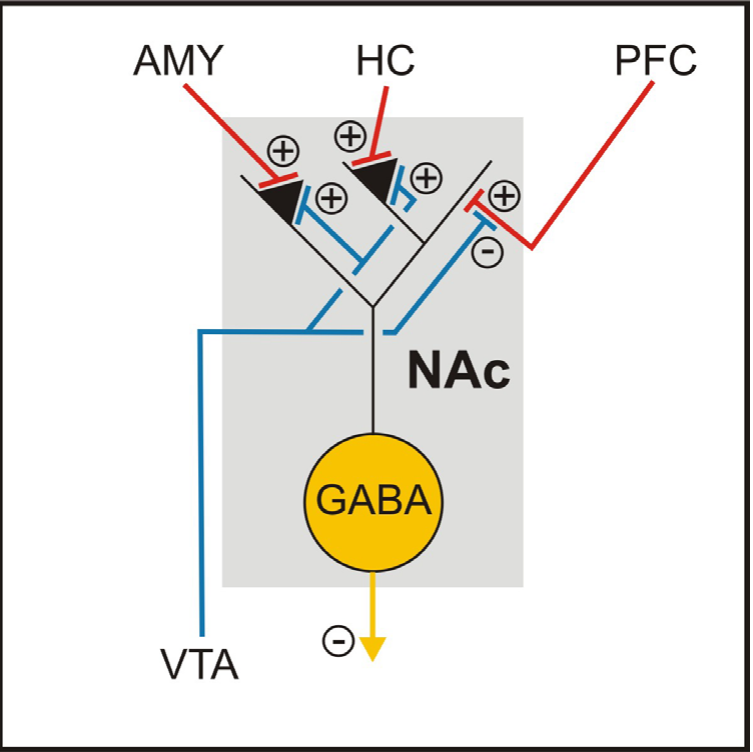
**Neuronal connections of Nucleus Accumbens**. Glutamatergic afferences from the prefrontal cortex, hippocampus and amygdala terminate on GABAergic medium spiny neurons and are modulated pre- and postsynaptically by dopamine. The glutamatergic input from the hippocampus and the amygdala drives the medium spiny neurons into a depolarized state and the input from the prefrontal cortex is capable of triggering action potentials [119]. AMY = amygdala; HC = hippocampus; PFC = prefrontal cortex; NAc = nucleus accumbens; VTA = ventral tegmental area.

There is an ongoing debate on the DAergic pathology of the NAc in ADHD (fig. 2). Both a lack and an excess of DA transmission seem to be supported by the available experimental evidence. The higher DAT density in ADHD patients, which should improve DA clearance from the synaptic cleft [28-30], the action of MPH as an indirect DA agonist, and imaging data demonstrating increased extracellular DA concentrations in the striata of healthy controls after MPH treatment [51], all argue for a reduced striatal DAergic transmission in ADHD. The opposing view assuming an accumbal DA hyperfunction in ADHD, in contrast, maintains that DAT density may be regarded as a measure of DA fibre density [31-34]. It further proposes the fact that there are two different kinds of DA transmission in the striatum [52]: Firing of DA neurons leads to a phasic release of DA in relatively high concentrations. The transmitter is cleared by the very effective DAT, so only very low concentrations of DA remain in the extracellular space. This tonic transmission is, however, still strong enough to activate autosynaptic D2 receptors which inhibit phasic DA firing. By blocking the DAT, MPH may increase the tonic extracellular DA concentrations and thus decrease the phasic transmission [53]. The observation that DA antagonists increase the positive effect of MPH on motor behaviour [54,55], but prevent its enhancement of cognitive capacities [56], further supports this hypothesis. In this view, the two impairments in PFC and NAc are probably even causally related, since alterations of DA metabolism in the PFC reciprocally change the DA activity in the striatum [57-62].

**Figure 3:**
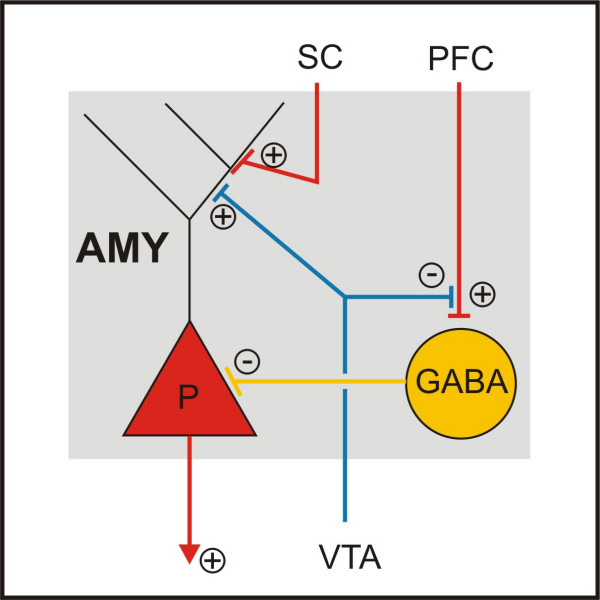
**Neuronal connections of Amygdala**. Glutamatergic afferents from the prefrontal cortex (PFC) contact GABAergic interneurons in the basolateral amygdala, which then inhibit the firing of pyramidal cells, while afferents from sensory cortices terminate mainly on glutamatergic pyramidal cells. The PFC input is suppressed presynaptically, whereas the sensory cortical input is enhanced postsynaptically by DA [67,120]. AMY = amygdala; P = pyramidal cell; PFC = prefrontal cortex; VTA = ventral tegmental area, SC = sensory cortices.

The DA projection to the amygdala matures in close coordination with that of the PFC, such that DA hypoinnervation of the PFC goes along with DA hyperinnervation of the amygdala (and entorhinal cortex) after early trauma [63]. Furthermore, the amygdala receives a strong projection from the PFC [64] which serves to put reflexive fear reactions under cognitive control (fig. 3) [65-67]. Although these neuronal effects after early trauma in gerbils should be considered only as a partial model of ADHD, it might be fruitful for further reasoning to remember that a high frequency of associated emotional problems has been reported in ADHD patients [9], but very little is known about DA function of the amygdala and its modifications by MPH in these cases.

### Therapeutic effects of methylphenidate

The most commonly used genetic rodent model of ADHD is the spontaneously hypertensive rat (SHR) [rev. in [68-70]]. In this model, reduced DA transmission was found in the PFC and striatum [7,71]. In the NAc, D1 receptor densities were increased, while D2 receptor densities were lowered [32,72-74] – which is in line with the current conception of ADHD in humans as outlined above. Oral MPH treatment for two weeks significantly changes these receptor densities to normal values [32,74]. Accordingly, Russell and colleagues [7] reported that MPH treatment alleviates ADHD-like symptoms in this rodent model.

In our lab, we studied the long-term plastic effects of MPH in a model of hyperkinetic behaviour that bears some resemblance to ADHD, i.e. gerbils after an early traumatic experience [33,34]. Early trauma is not a typical, but a possible factor in the aetiology of ADHD [75]. A single high dose of methamphetamine (MA), administered on postnatal day 14, causes a syndrome in young-adult gerbils that is characterised by hyperactivity, increased fearfulness and impaired PFC function in both working memory and extinction [76,77]. Neuroanatomically, this is based on decreased prefrontal and accumbal, but increased entorhinal and amygdalar DA fibre densities [63,78,79]. Other neuromodulators like serotonin and acetylcholine adapt to this changed situation by altering their innervation densities, too [80,81].

In this animal model of early traumatic experience, we applied MPH both orally and (see below) intraperitoneally (i.p.) during adolescence (PD30–60) to isolation-reared, MA intoxicated gerbils. While the oral application was designed to match human medication, the i.p. application was meant to study the effects of MPH abuse. Two control groups were taken, one being left undisturbed, whereas the other received water. DA fibres were stained immunohistochemically, and their densities assessed by computerised image analysis in the PFC (anterior cingulate, prelimbic and infralimbic), ventral striatum (NAc core and shell, olfactory tubercle) and the central (lateral and medial) and basolateral amygdalar subnuclei. Additionally, cell proliferation rates in the hippocampal dentate gyrus were counted as a measure of long-term memory plasticity.

**Figure 4:**
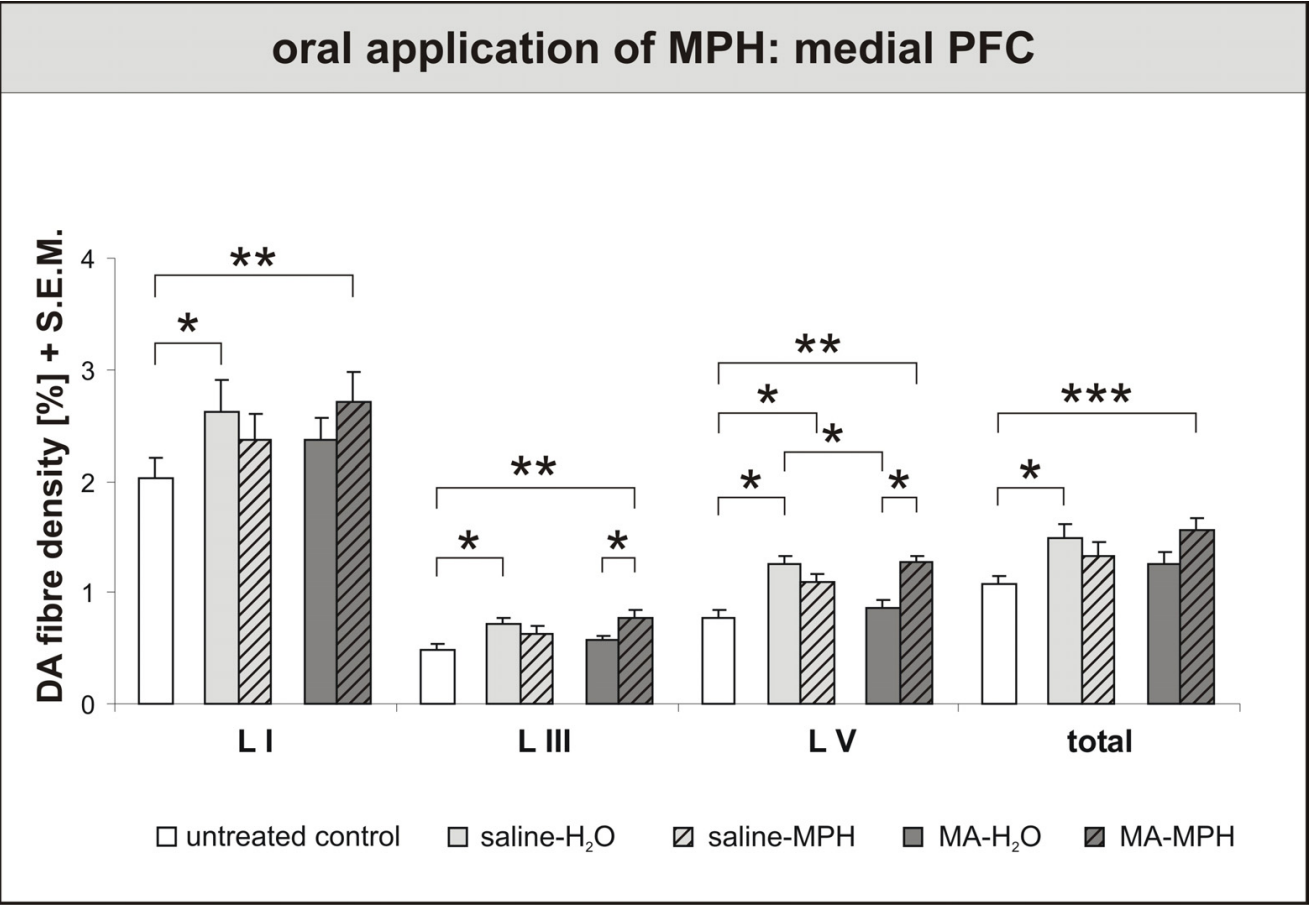
**Oral application of Methylphenidate (MPH) – Effects on Prefrontal Cortex (PFC)**. Dopamine (DA) fibre density + S.E.M. is presented in lamina I, III and V of the PFC. Three effects are noteworthy. First, methamphetamine (MA) (= MA-H_2_O) impaired the maturation of DA fibres in layer V, as had been shown before [78]. Second, MPH treatment for 30 days returned DA fibre densities to control values in MA-traumatised (= MA-MPH) animals. In control animals, in contrast, MPH (= saline-MPH) did not change the DA fibre densities, or even rather reduced them. Third, application of water (= saline-H_2_O), i.e., pure handling, was highly effective in increasing the DA fibre densities in all layers. As isolated rearing by itself allows only for a suppressed maturation of DA fibres, this latter finding suggests that handling is a beneficial, "therapeutic" intervention (Lehmann, Grund et al., unpublished observations). For biostatistics two-way ANOVA with post-hoc contrast analysis among treated groups or pairwise comparisons with t-tests for untreated controls vs. treated groups were used for each lamina; significance values: *p < 0,05, **p < 0,01, ***p < 0,001.

In the PFC, three effects are noteworthy (fig. 4). First, MA (= MA-H_2_O) impaired the maturation of DA fibres in the prelimbic cortex, as had been shown before [78]. Second, MPH treatment for 30 days returned DA fibre densities to control values in MA-traumatised (= MA-MPH) animals. In control animals, in contrast, MPH (= saline-MPH) did not change the DA fibre densities, or even rather reduced them. Third, application of water (= saline-H_2_O), i.e., pure handling, was highly effective in increasing the DA fibre densities in both the anterior cingulate and prelimbic cortices. As isolated rearing by itself allows only for a suppressed maturation of DA fibres, this latter finding suggests that handling is a beneficial, "therapeutic" intervention (Lehmann, Grund et al., unpublished observations).

**Figure 5:**
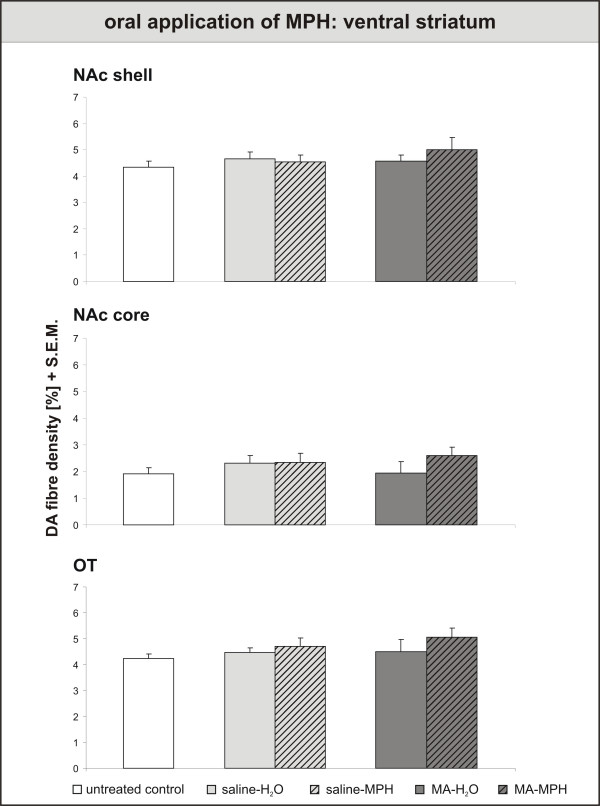
**Oral application of Methylphenidate – effects on Ventral Striatum**. Dopamine (DA) fibre density + S.E.M. is presented in the nucleus accumbens shell, core and olfactory tubercle. In the nucleus accumbens, neither handling nor MPH exerted any effects on the DA innervation of the ventral striatum. For abbreviations, see fig. 4. Biostatistics: ANOVA with repeated measures and t-tests (see fig. 4).

**Figure 6:**
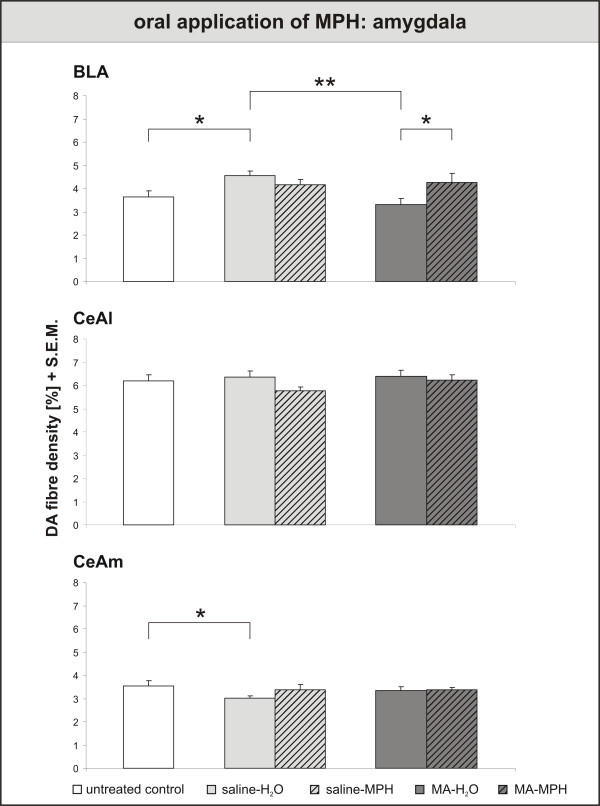
**Oral application of Methylphenidate – effects on Amygdala**. Dopamine (DA) fibre density + S.E.M. is presented in the basolateral, lateral and medial central amygdala. In the basolateral amygdala, results correspond to those in the PFC: Handling-induced rise in DA fibre density, MA-induced decrease and MPH-induced recovery. The DA innervation of the central amygdala did not react to MPH, but was lowered in the medial part by handling. For abbreviations, see fig. 4. Biostatistics: ANOVA with repeated measures and t-tests (see fig. 4); significance values: *p < 0,05, **p < 0,01.

In contrast to the PFC results, neither handling nor MPH exerted any effects on the DA innervation of the ventral striatum (fig. 5). If anything, there seemed to be a slight, but non-significant rise in fibre densities after MPH treatment in MA-intoxicated animals. In the basolateral amygdala (fig. 6), results correspond to those in the PFC: Handling-induced rise in DA fibre density, MA-induced decrease and MPH-induced recovery. The DA innervation of the central amygdala did not react to MPH, but was lowered in the medial part by handling.

That MPH restores the DA innervation of the gerbil PFC that was lesioned by MA indicates a beneficial effect of MPH treatment, which is confirmed by studies demonstrating improved attention and working memory in animals treated with MPH [82,83]. Since no impairments in long-term memory have been found in ADHD patients [84], and MPH, consequently, does not seem to influence this neuropsychological function [85-88], it is not surprising that we did not detect any effect of MPH on the hippocampal cell proliferation (data not shown). As numerous endo- and exogenous substances, including many psychopharmacological drugs, have been demonstrated to alter the dentate mitotic activity [rev. in [89], this result rather underlines the specificity of MPH as an enhancer of PFC/NAc-based function.

Concerning the clinical use of MPH, the reported findings in animals suggest that disturbed brain systems may react differently to the drug than those with a normal development. However, empirical evidence in humans with and without ADHD shows that the situation is rather complex. At the clinical behavioural level, Rapoport and Inoff-Germain [90] reported in an update that stimulants appear to have basically similar behavioural effects in normal and in hyperactive children, as had first been shown for dextroamphetamine [91,92]. But taking the neuropsychological level of behaviour into account, a different picture can be seen: MPH improved response inhibition in the healthy and ADHD group in one task and only in ADHD children in the other task [93]. Similarly, Elliott et al. [94] found that MPH influenced performance in two conflicting ways in healthy young adults; enhancing executive aspects of spatial function in novel tasks but impairing previously established performance. Further, beneficial effects of MPH on working memory seem to be greatest in the subjects with lower baseline working memory capacity [95]. Finally, looking at neural substrates of motor and cognitive control in fMRI, MPH seems to affect striatal activation differently in ADHD (positive) than in healthy (negative) children while increased frontal activation was seen in both groups [93]. A neurophysiological study with transcranial magnetic stimulation could demonstrate opposite effects of MPH on neuronal excitability in children with ADHD versus healthy controls [96].

In conclusion, a transfer from animal data on MPH to MPH treatment in human ADHD is always limited. Although the neuronal systems of ADHD patients work differently than those of healthy controls, some performing/behavioural output after MPH looks similar. However, distinct rather than unitary patterns of functional abnormality in ADHD have to be taken into consideration and a differential treatment approach seems to be adequate, which might work best for patients with an unequivocal ADHD symptomatology and psychosocial impairment, although only strong levels of response may be predicted by a few baseline characteristics (e.g. considerable inattentiveness [97]).

Against this backdrop, our results rather support the notion that psychomotor impaired individuals and healthy controls show indeed opposite responses to ADHD (fig. 4). This finding is further corroborated by behavioural animal studies which show that MPH treatment improves attention in bad performers, but has no effect on normal controls in the 5-choice serial reaction time task [82]. Furthermore, MPH did not induce locomotor sensitization in SHRs [98], but caused both locomotor sensitization and cross-sensitization to amphetamine in normal rat strains [98,99]. More studies will be needed to clarify this issue.

Although we did not find altered DA fibre densities in the NAc, MPH treatment indeed exerts significant and long-lasting functional effects in the NAc: In contrast to other stimulant drugs, MPH does not sensitise the rewarding effects of other drugs, but instead reduces the risk for substance abuse both in rats [100,101] and humans [[102], rev. in [103]], although there are conflicting results in rats [104]. Since clinical doses do not increase extracellular accumbal DA levels, but change noradrenaline concentrations [105-107], it seems likely that this beneficial effect is not mediated primarily via the DA system. Nevertheless, as mentioned above, accumbal DA receptor densities are altered by MPH treatment [32]. Furthermore, MPH treatment during adolescence lastingly decreases the DAT concentration in the NAc, while leaving the densities of the serotonin and noradrenaline receptors untouched [20]. Thus, the alterations occuring in the NAc are obviously rather of a physiological kind, possible due to the much earlier maturation of the accumbal than the prefrontal DA innervation [12,108,109].

The increased DA fibre density found in the basolateral amygdala of MPH-treated MA-intoxicated gerbils is a first hint that MPH affects neural systems beyond PFC and NAc. It corresponds to further results from the above mentioned behavioural study showing that MPH-treated rats respond stronger to aversive situations and show more anxiety-like behaviour [101].

### Methylphenidate as a psychostimulant drug of abuse

Orally taken, MPH has no abuse potential because of its slow increase of the plasma level, while a "high" (with the associated risk of drug abuse) can be elicited by i.v. application [110]. Hence, when abused, MPH is usually applied intravenously [103,111]. This route of application dodges the pronounced hepatic first-pass metabolism that MPH is subjected to after oral consumption [112]. In consequence, higher plasma concentrations are reached about six times faster and with a much shorter half life [113], and extracellular DA concentrations in the NAc are higher [106]. Although the abuse of MPH is rather rare, it seems to be important to investigate if the potential long-term plastic effect of this kind of application differs from that of oral application.

To our knowledge, there is as yet no other animal study on the long-term effects of intraperitoneally or intravenously applied MPH except for one from our lab that we briefly summarise here [33,34]. We studied the plastic long-term effects of i.p. MPH on the DA innervation in the above described model. Two different concentrations of MPH were investigated, one (5 mg/kg) in the clinical range, the other (50 mg/kg) clearly beyond it. DA fibre densities were measured in the ventral striatum and amygdala. Data for the PFC could not be obtained out of technical problems, but previous experience with our model would suggest that DA fibres in the PFC react in a similar way to those in the NAc.

**Figure 7:**
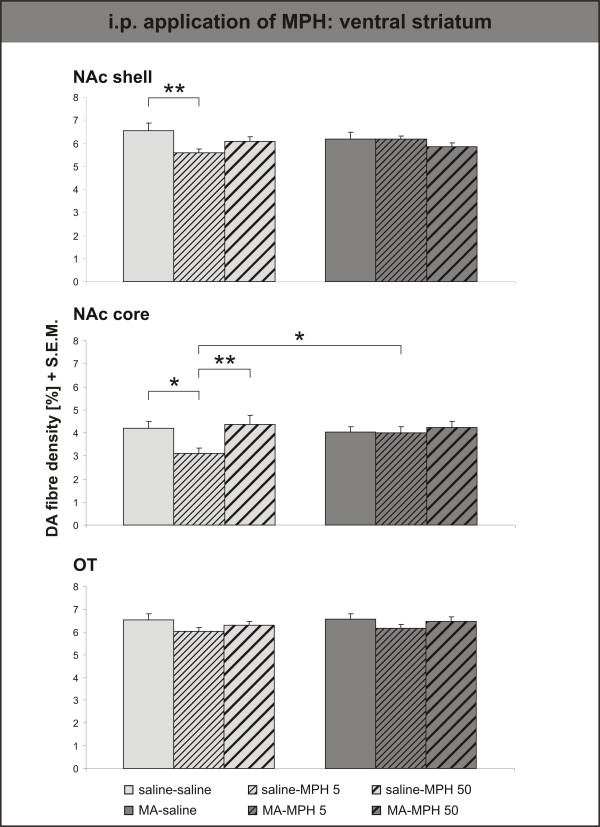
**Intraperitoneal application of Methylphenidate – Ventral Striatum**. Dopamine (DA) fibre density + S.E.M. is presented in the nucleus accumbens (NAc) shell, core and olfactory tubercle. I.p. MPH only had an effect in control animals, and only the lower, clinical dose (= saline-MPH 5) was effective in reducing the DA innervation density in both subterritories of the NAc. The DA innervation was unaltered by the higher dose of MPH (= saline-MPH 50), and even significantly denser than after treatment with the clinical dose in the NAc core. For abbreviations, see fig. 4. Biostatistics: Two-way ANOVA with repeated measures; significance values: *p < 0,05, **p < 0,01.

**Figure 8:**
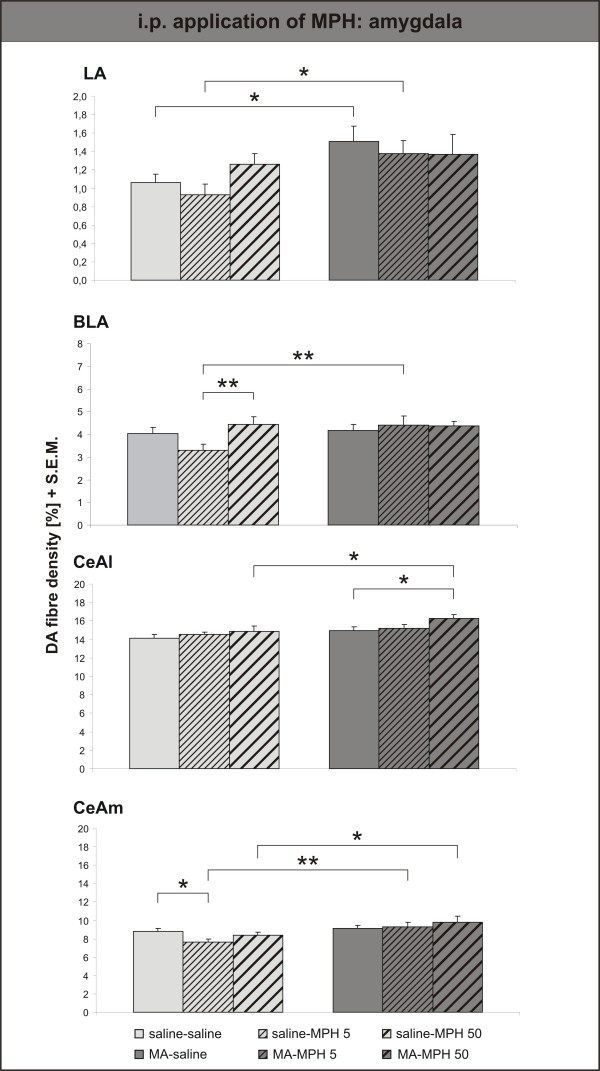
**Intraperitoneal application of Methylphenidate – Amygdala**. Dopamine (DA) fibre density + S.E.M. is presented in the lateral, basolateral, lateral and medial central amygdala. Similar, albeit not quite significant effects were observed in the lateral, basolateral and medial part of the central amygdala. Only in the lateral part of the central amygdala did the high dose of MPH (= MA-MPH 50) i.p. increase the DA fibre density in MA-intoxicated animals. For abbreviations, see fig. 4. Biostatistics: Two-way ANOVA with repeated measures; significance values: *p < 0,05, **p < 0,01.

Surprisingly, i.p. MPH only had an effect in control animals, and only the lower, clinical dose (= saline-MPH 5) was effective in reducing the DA innervation density in both subterritories of the NAc (fig. 7). The DA innervation was unaltered by the higher dose of MPH (= saline-MPH 50), and even significantly denser than after treatment with the clinical dose in the NAc core. Similar, albeit not quite significant effects were observed in the lateral, basolateral and medial part of the central amygdala (fig. 8). Only in the lateral part of the central amygdala did the high dose of MPH (= MA-MPH 50) i.p. increase the DA fibre density in MA-intoxicated animals.

Our results demonstrate that by i.p. application, MPH can indeed impair the postnatal maturation of DA fibres in the ventral striatum in a similar way as a single high dose of MA [79,109]. We can offer no explanation why the higher dose of MPH has no detectable effects in the anatomical dimension. It has been shown, however, that already a single dose of MPH in that range (30 mg/kg) sensitises rats for the locomotor-effect of amphetamine [105], thus making them more prone for a drug addiction. An up-regulation of the cAMP pathway in the NAc mediated via D2 receptors has been implicated in this kind of sensitization [114], so here again changes may rather be intracellular and physiological than anatomical.

## Conclusion

MPH is an indispensable drug that beautifully fits the pharmacological demands to regulate DA dysfunctions in ADHD. In patients with this disorder, MPH simultaneously compensates a prefrontal DA hypofunction and probably restrains an accumbal DA hyperfunction in the long run. Animal studies suggest that this effect is supported in the PFC by enhancing the maturation of DA fibres [33,34], whereas adaptations of pre- and postsynaptic receptor densities are elicited in the NAc. Both clinical and preclinical studies converge to confirm that in subjects suffering from cognitive-motivational and neural impairments, MPH has long-term beneficial effects in several respects, e.g. by reducing the core symptoms of ADHD as well as the risk for substance abuse [e.g. [102,103,115]. A certain reservation must be deduced from the observation that both the DA innervation and the behavioural function of the amygdala are altered by MPH, making animals more fearful and sensitive to stressful stimuli [33,101]. However, since the behavioural study used normal rats, further investigations are needed to check whether adverse emotional effects are also evoked by MPH in animal models of ADHD.

This latter consideration directly leads us to one of two important caveats concerning the use of MPH: Behavioural studies show that MPH is ineffective in rodents without attentional impairments [82], as far as attention is concerned. In contrast, MPH elicits locomotor sensitization in non-hyperactive rat strains, whereas it has no such effect in SHRs [98]. The assessment of DA fibre densities confirms that these are only improved in previously traumatised animals, but unchanged or possibly even reduced in healthy controls [33,34]. Transformed to a clinical perspective, this might suggest that physicians are possibly dealing with (at least partly) quantitatively and/or qualitatively different responder systems when treating the brains of children with or without ADHD [see also [7,116]]. This perspective is supported by different effects of MPH on neuronal excitability (measured with transcranial magnetic stimulation) in healthy persons compared with ADHD patients [96]. However, as discussed above, there are also partly conflicting data [90-95], making it impossible to arrive at a firm conclusion so far.

Finally, being a psychostimulant, MPH has unfortunately also been discovered by some as a drug of abuse that is intravenously applied. First results on the long-term effect of such abuse in animals has shown equivocal results, with negative effects similar to methamphetamine in low but no effect with high doses of MPH [33,79]. It remains to be checked whether it may even be neurotoxic under such conditions. Nevertheless, the wealth of human and animal information on MPH shows the great value of the drug which has to be handled with care to use it in the right way.

## Competing interests

The author(s) declare that there are no competing interests.

## Authors' contributions

All authors are involved in the idea and planning of the reported own studies as well as in the writing of the manuscript. The Bielefeld authors conducted the reported own animal experiments in their laboratory.
